# Syncope Recurrence and Downstream Diagnostic Testing after Insertable Cardiac Monitor Placement for Syncope

**DOI:** 10.3390/diagnostics12081977

**Published:** 2022-08-16

**Authors:** Camille G. Frazier-Mills, Lawrence C. Johnson, Ying Xia, Sarah C. Rosemas, Noreli C. Franco, Sean D. Pokorney

**Affiliations:** 1 Division of Cardiology, Duke University School of Medicine, Durham, NC 27710, USA; 2 Medtronic, Inc., 200 Coral Sea St., Mounds View, MN 55112, USA

**Keywords:** insertable cardiac monitor, implantable loop recorder, recurrent syncope, diagnostic tests, real-world claims data

## Abstract

Despite advances in syncope evaluation strategies and risk stratification, the high cost of syncope is largely driven by extensive and often repetitive testing. This analysis of a large deidentified US claims database compared the use of diagnostic tests, therapeutic procedures, and the recurrence rate of acute syncope events before and after placement of an insertable cardiac monitor (ICM) in syncope patients. The patients had a minimum of 1 year of continuous enrollment before and 2 years after ICM placement. Among 2140 patients identified, a statistically significant reduction in the use of 14 out of 18 tests was observed during follow-up compared with pre-ICM testing. During the 2-year follow-up, 28.3% of patients underwent cardiac therapeutic interventions after a median of 127 days. Significantly fewer patients experienced acute syncope events during the 1st and 2nd years of ICM follow-up compared with the 1-year pre-ICM period, and the frequency of events per patient also decreased. In conclusion, reductions in diagnostic testing and acute syncope events were observed after ICM placement in a large real-world cohort of unexplained syncope patients. Further studies are needed to prospectively assess the impact of ICM vs. short-term monitoring on patient outcomes and healthcare utilization.

## 1. Introduction

Syncope accounts for 1% of all emergency room evaluations and results in 1–6% of all hospital admissions in the Unites States [1,2]. In 2000, the estimated annual cost for syncope hospital admissions was $2.4 billion USD, driven largely by extensive and often repetitive testing [3]. Despite advances in the risk stratification and evaluation of syncope, the rates of syncope-related ED (emergency department) visits, hospital admission diagnoses, and hospital discharge diagnoses have not changed significantly over a 10-year period [4].

There is broad variation in the diagnostic yield of various tests and procedures for the diagnosis of syncope. Insertable cardiac monitors (ICMs) are associated with higher rates of symptom-rhythm correlation relative to Holter or event monitors, and recent clinical guidelines for syncope recommend the use of ICMs early in the evaluation of unexplained syncope [1,5].

Few publications have focused on syncope event recurrence and the rates of downstream diagnostic testing among patients with an ICM. The first objective of this analysis was to assess the rates of use for various diagnostic tests pre-ICM and during ICM follow-up using a large claims data set. The second objective was to determine the time from ICM placement to therapeutic intervention (pacemaker, implantable cardioverter-defibrillator, or ablation). We also compared the recurrence rate of acute syncope events between 1-year pre-ICM and the subsequent 2 years of follow-up.

## 2. Methods

### 2.1. Data Source

This analysis utilized administrative claims data from the 2008–2016 Optum deidentified Clinformatics^®^ Data Mart Database. The Optum database includes approximately 17–19 million annual covered lives, from both commercial and Medicare Advantage health plans [6]. Based on the Health Insurance Portability and Accountability Act, the use of deidentified data does not require institutional review board approval or a waiver. The data elements used for this analysis included demographic data; enrollment information; hospital admission and outpatient encounter dates; International Classification of Diseases—9th Edition—Clinical Modification (ICD-9-CM), 10th Edition (ICD-10-CM), ICD-9-PCS (Procedure Classification System), and ICD-10-PCS codes; Current Procedural Terminology (CPT) codes; and the date, amount, and medication type for prescription fills.

### 2.2. Study Population

**Figure 1:**
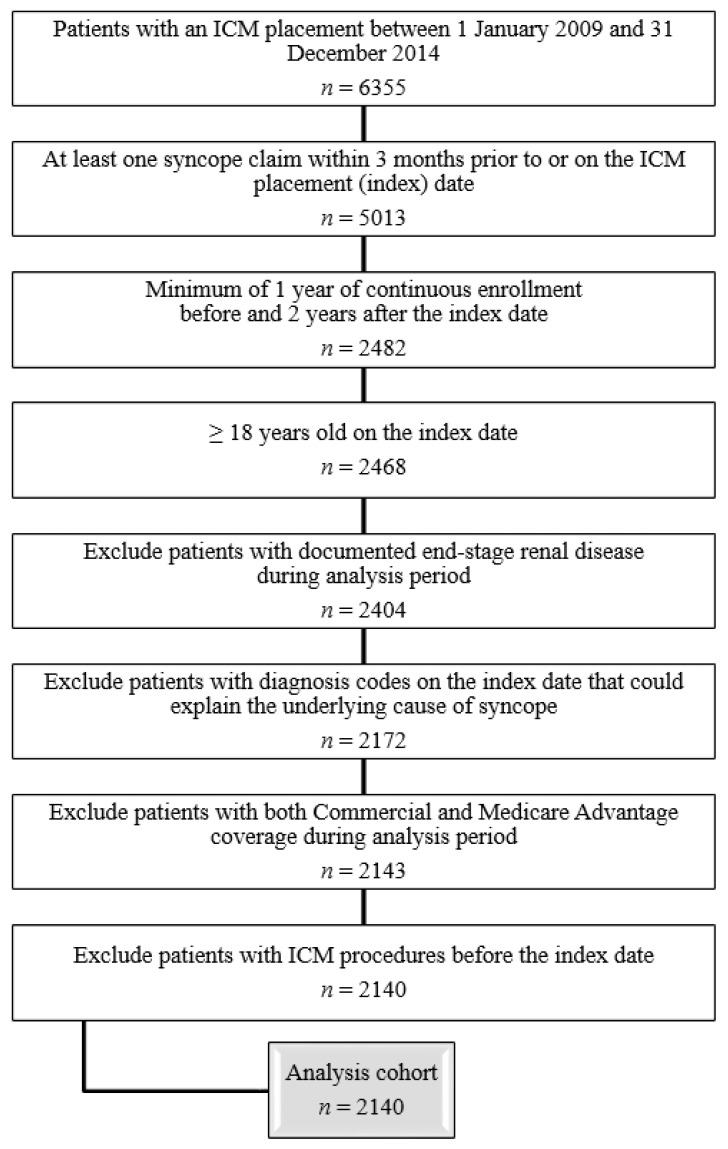
Consort diagram. Abbreviation: ICM: Insertable cardiac monitor.

Patients ≥18 years of age who received an ICM between 1 January 2009 and 31 December 2014 were included in the analysis (Figure 1), with the ICM insertion date serving as the index date. Patients were required to have a history of syncope, defined as a diagnosis code for syncope in any care setting (inpatient, outpatient, or office-based). This syncope diagnosis code was required to be coded on or within 3 months prior to ICM placement, in order to increase the likelihood that the ICM was placed for a syncope. Additionally, the included patients had a minimum of 1 year of continuous enrollment before and 2 years after the index date. Patients were excluded if they had any of the following criteria: documented end-stage renal disease during the analysis period, an ICM implanted prior to the index date, or both commercial and Medicare Advantage coverage during the study period. Patients with diagnosis codes on the index date that could explain the underlying cause of syncope (such as blood volume depletion, neurological conditions, end-stage renal disease, Parkinson’s disease, and anemia, among others (Table S1)) were excluded to capture only the unexplained syncope population.

### 2.3. Study Outcomes

The number of imaging studies, rhythm monitoring tests, laboratory testing, and electrophysiology studies that were performed 1-year pre-ICM and during 2 years of follow-up were determined through CPT codes. Similarly, the number of ablation procedures, pacemaker implants, and automatic implantable cardioverter-defibrillator (AICD) implants were determined during the 2-year ICM follow-up period. Acute syncope events during the 1-year pre-ICM period and the 1st and 2nd years of ICM follow-up were identified based on the presence of a syncope diagnosis code (ICD-9-CM 780.2 or ICD-10-CM R55) at an acute site of service (inpatient hospital, emergency department, or urgent care). Syncope claims reported at other places of service, such as a physician office, were not considered as acute syncope events. Only unique encounters on separate dates from inpatient hospital, emergency, or urgent care settings were included to ensure that multiple claims were not assigned to a single syncope event. Syncope-related injuries were identified based on the presence of an injury diagnosis code during an acute syncope event (Table S2).

### 2.4. Statistical Analysis

To determine the rate of recurrent syncope events after the index date, the following were calculated: means, medians, standard deviations (SD), interquartile ranges, minimums, and maximums. Descriptive analyses were conducted for the characteristics and clinical variables during 1 year pre-ICM and the follow-up period. In addition, negative binomial and binomial models were conducted to calculate *p*-values for continuous variables and binomial variables, respectively, to determine whether there were statistically significant differences among recurrent syncope events and the utilization of diagnostic tests pre-ICM and during follow-up. The selection of negative binomial models was driven by observed overdispersion in the count-related outcomes. All statistical tests were two-sided and were considered significant at the alpha level of 0.05 (*p*-value < 0.05). All analyses were performed using SAS version 9.4 (SAS, Raleigh, NC, USA).

## 3. Results

In total, 6355 patients received an ICM between 2009 and 2014, and 5013 of those patients had a claim containing a syncope diagnosis code (ICD-9 diagnosis code 780.2: syncope and collapse) at any site of service on or within 3 months prior to ICM insertion (Figure 1). After applying all other inclusion and exclusion criteria, the analysis cohort included 2140 unexplained syncope ICM patients (Figure 1). The patients had a median age of 73 years (25–75th% = 62.0–81.0 years), 54% (*n* = 1156) were female, and they had a median Charlson Comorbidity Index of 2.0 (25–75th% = 1.0–3.0) (Table S3). Two-thirds of the patients (*n* = 1427) had Medicare Advantage plans. The most frequent comorbidities were hypertension (77.8%), coronary artery disease (38.2%), diabetes (25.0%), COPD (chronic obstructive pulmonary disease), asthma (15.1%), and seizures (14.4%). There were 396 patients (18.5%) who experienced previous injuries related to syncope.

### 3.1. Diagnostic Testing and Health Care Utilization

**Figure 2:**
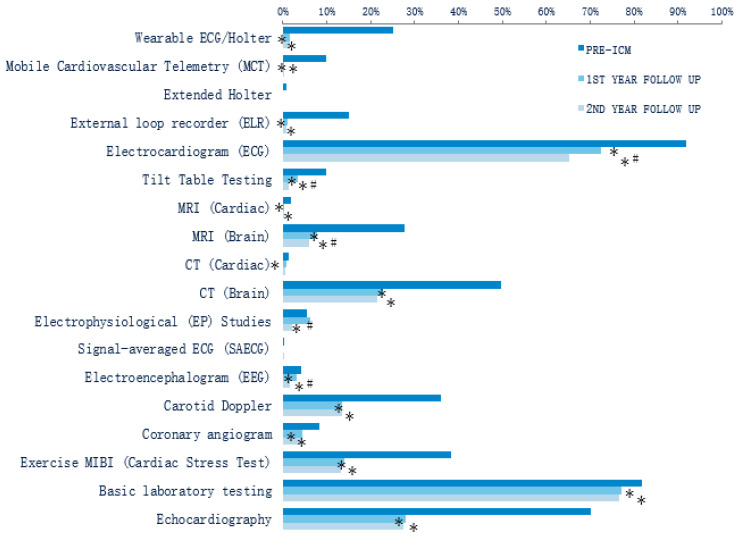
Diagnostic tests used in an attempt at clinical diagnosis (whole cohort; *n* = 2140). * *p* < 0.05 when either the 1st-year or 2nd-year follow-up were compared to pre-ICM; # *p* < 0.05 when the 2nd year of follow-up was compared to the 1st year of follow-up.

A total of 18 diagnostic tests that are frequently used for the evaluation of patients with unexplained syncope were compared between the pre-ICM and follow-up periods (Figure 2). The overall rates of testing across all 18 tests were numerically lower in the first year of follow-up compared with pre-ICM (Figure 2). Statistically significant reductions in the rates occurred in 14 out of 18 (77.8%) testing modalities. Similarly, there were numerically lower rates of all the diagnostic tests in the second year of follow-up, compared with pre-ICM, with 16 out of 18 (88.9%) tests having statistically significant lower rates. When comparing the tests used between the first and second years of follow-up, the proportion of patients who had an electrocardiogram (ECG) (72.5% vs. 65.2%, *p* < 0.001), tilt table test (3.4% vs. 1.2%, *p* < 0.001), magnetic resonance imaging (MRI) of the brain (7.7% vs. 6.0%, *p* < 0.001), electrophysiology study (EPS) (6.3% vs. 2.1%, *p* = 0.016), or electroencephalogram (EEG) (3.0% vs. 1.6%, *p* < 0.001) was statistically lower in the second year. The ambulatory external cardiac monitor use (including ECG/Holter, extended Holter, mobile cardiac telemetry, and external loop recorder) remained low (2.6%) during the second year of follow-up and was not significantly different compared with the first year (3.1%).

During the 2-year follow-up period post-ICM, repeated use of the same diagnostic test was rare (<5% of patients) for all tests except: ECG (71.2% of patients with ≥ two tests), computed tomography (CT) of the brain (21.4%), carotid Doppler (8.7%), cardiac stress test (13.7%), basic laboratory testing (39.6%), and echocardiography (21.8%); Table S4.

Diagnostic testing was also assessed for the subset of patients with an acute syncope event (i.e., syncope coded in the emergency room or inpatient setting) during the pre-ICM period (*n* = 885). Consistent with our broader patient population of patients with a general history of syncope (syncope coded in any care setting), a decrease in diagnostic testing between pre-ICM and the subsequent 2 years of follow-up was also found in those with prior acute events (Figure S1).

Diagnostic test utilization for the three study periods was compared between patients with (*n* = 885) vs. those without (*n* = 1255) an acute syncope event during the pre-ICM period. Patients who had an acute syncope event had significantly more tests performed than those without in the 12 months pre-ICM (ECG, tilt table, MRI of the brain, CT of the brain, EPS, EEG, carotid Doppler, coronary angiogram, cardiac stress test, basic laboratory testing, and echocardiography; Table S5). Only a wearable ECG/Holter was more frequently used in patients without acute syncope (Table S5). In contrast, during the first and second years of follow-up, there were few differences in diagnostic test utilization between patients with vs. without acute syncope events during the pre-ICM period (Tables S6 and S7).

### 3.2. Therapeutic Interventions in Follow-Up

**Figure 3:**
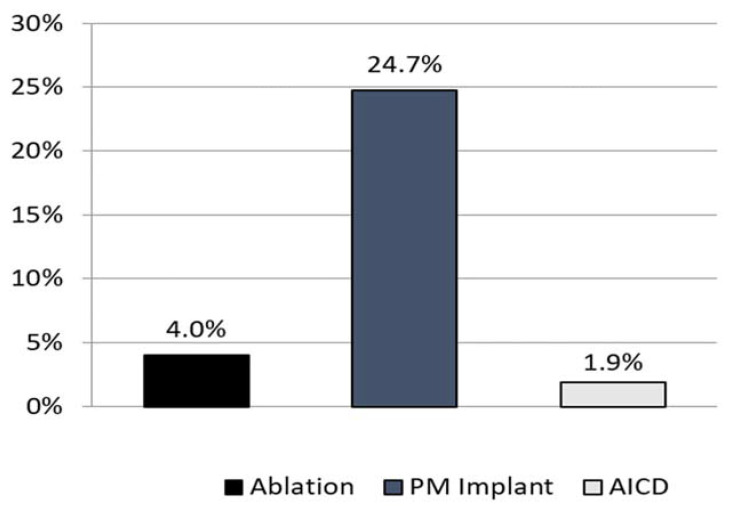
Therapeutic interventions over the 2-year follow-up period. Abbreviations: PM: pacemaker; AICD: automatic implantable cardioverter-defibrillator.

A total of 606 (28.3%) patients underwent 646 therapeutic interventions during the 2-year follow-up period, with a median (IQR) time to treatment of 127 (50–324) days (Table 1). A pacemaker implant (24.7%) was the most frequently performed procedure, followed by a cardiac rhythm ablation (4.0%) and AICD implant (1.9%) (Figure 3). The three most common diagnoses for subsequent ablation in this syncope cohort were atrial fibrillation (32.8%), other specified cardiac dysrhythmia (17.9%), and paroxysmal supraventricular tachycardia (11.9%). The three most common diagnoses for a pacemaker were sinoatrial node dysfunction (26.5%), syncope and collapse (18.6%), and other specified cardiac dysrhythmia (9.3%), while complete heart block was present in 2.7% patients. The most common diagnosis for an AICD implant was paroxysmal ventricular tachycardia (28%).

### 3.3. Syncope Event Rate during Follow-Up

The proportion of patients experiencing at least one acute syncope event was higher pre-ICM (*n* = 885, 41.4%) compared with the first year (*n* = 492, 23.0%) and second year (*n* = 147, 6.9%) of follow-up (*p* < 0.001 for all). This represented a decrease in the syncope events of 44.4% for the first year of follow-up and 83.4% for the second year compared with pre-ICM. Moreover, there was a drop in the recurrent syncope events of 70.1% during the second year compared to the first year of follow-up. In addition to a lower proportion of patients experiencing acute syncope events, the mean ± SD number of syncope events per patient was also lower in the first (1.1 ± 3.0) and second (0.3 ± 1.3) years of follow-up compared with pre-ICM (2.3 ± 4.2) (*p* < 0.001 for all). Of the three time periods studied, the pre-ICM period had the greatest variation in terms of the number of syncope events per patient, as evidenced by the relatively high SD.

## 4. Discussion

The results from this claims database analysis showed that, among commercial and Medicare Advantage patients receiving an ICM for an unexplained syncope, fewer imaging studies and diagnostic tests were conducted after ICM placement compared with prior to ICM. More than one in four patients (28.3%) had an electrophysiology intervention (ablation, pacemaker, or AICD) performed within 2 years of ICM follow-up. Recurrent syncope rates reported in acute sites of service were lower over time after ICM placement compared with pre-ICM.

The syncope guidelines in the United States from the American College of Cardiology/American Heart Association/Heart Rhythm Society [1] and the syncope guidelines in Europe from the European Society of Cardiology/European Heart Rhythm Association [5] both provide decision support algorithms that can be utilized for patient management. The use of an ICM in unexplained syncope that is not frequent enough that a short-term monitor would allow a symptom–rhythm correlation is recommended in the decision support within both guidelines (class IIa in the American and class I in the European guidelines). Moreover, previous analyses have demonstrated that the consistent use of decision support algorithms results in lower health care utilization [7,8]. The current analysis found that the rate of diagnostic testing was substantial during the year prior to ICM insertion; this is consistent with the results of the PICTURE registry [9], which found that a median of 13 diagnostic tests had been performed prior to ICM insertion. The rates of diagnostic testing in our study significantly decreased during the first and second years of ICM follow-up for 14 out of 18 diagnostic tests relative to the year pre-ICM. This decrease remained consistent when evaluating diagnostic tests used specifically among patients with a syncope during the year pre-ICM. Although testing decreased markedly after ICM insertion, a small number of patients were observed to receive short-term external cardiac monitors (Holter, MCT, or ELR): 3.1% and 2.6% in years 1 and 2 post-ICM, respectively. We hypothesize that the use of external monitors could take place if providers choose to use a multi-lead monitor to better discern the rhythm and/or morphology, as well as to quantify the arrhythmia burden (PAC count and PVC count).

Acute syncopal events were frequently reported during the pre-ICM period, with two out of five patients experiencing events and an overall average of 2.3 events per patient. In contrast, the number of acute care visits for a syncope was significantly lower during the first (23%) and second (7%) years of follow-up and corresponded to a drop of 83% in recurrent syncope by the second year compared to the pre-ICM period. Our results are comparable to a different claims database analysis from the UK National Health Service, which showed that, among patients with two or more hospitalizations due to syncope within 2 consecutive years (mean age: 73 years), 25% were hospitalized for syncope within the next few months and 9% were hospitalized for an injury [10]. The latter also showed that, among syncope patients monitored for 3 years with an ICM, hospitalizations due to syncope dropped by 60% compared to the pre-ICM levels.

The 23% rate of syncope recurrence observed 1 year after ICM was lower than the 38% reported by the PICTURE registry [9]. However, variations between these two patient populations may explain the difference observed. Prior to ICM implant, 70% of patients in PICTURE had been hospitalized at least once for syncope, compared with 41% of the patients in our cohort with acute events in the year pre-ICM. This suggests that patients identified in the present analysis were prone to a lower frequency of syncope events than those in PICTURE. In addition, patients in PICTURE were younger (mean age 61 compared to a median age of 73 in the current analysis) and healthier than in our cohort. Another previous report of a non-ICM population that was more consistent with our cohort found that high-risk patients who were elderly and institutionalized had a 30% risk of syncope recurrence [11].

Therapeutic interventions were common after ICM placement for unexplained syncope, as more than 28% of the patients in our cohort underwent an intervention within 2 years of ICM follow-up. This is consistent with data from several studies reporting that approximately one in four syncope patients monitored by ICM for 1–3 years receive a pacemaker, implantable cardioverter-defibrillator, or have an ablation performed [9,12,13]. The reduction in acute events observed during the follow-up was presumably driven by the administration of therapeutic interventions.

Given the use of claims data in the current analysis, the diagnostic yield of the ICMs in our cohort was not known. However, a meta-analysis grouping 49 studies showed that the diagnostic yield of ICMs in unexplained syncope was 44% after 1 year of monitoring [14].

This analysis had several important limitations. Unexplained syncope has no distinct diagnostic code; therefore, unexplained syncope patients were identified based on the diagnosis code for syncope only in the absence of other diagnoses for explained syncope. Likewise, there are no specific codes differentiating vasovagal/reflex syncope vs. other forms of syncope so these patients could not be conclusively ruled out via claims-based coding. Due to this challenge in diagnostic coding, we could not include an appropriate comparator/control group to analyze the differences in outcomes in unexplained syncope patients without an ICM. There were also limitations in the level of detail in the claims database, so we were not able to reliably characterize patient diagnoses during follow-up, especially the arrhythmias that were noted on the ICMs at the times of recurrent events. While pacemaker implantation in reflex syncope has gained interest in clinical practice, we were unable to characterize this due to the lack of a diagnosis code for reflex syncope; this would be an interesting direction for future study. In this study, we characterize all diagnostic testing received in a population of unexplained syncope patients; however, as we cannot rely on the presence or absence of condition-specific diagnosis codes on each diagnostic testing claim, there could be reasons other than syncope for some of the testing observed.

The ICM does allow patients to remotely send rhythm data to their providers after a syncope event without having to go to an acute care setting. However, given the limitations in the available claims data, it was not clear whether acute care visits were lower over time because the care of these patients was shifted to the outpatient setting and the reassurance provided by the ICM or the impact of therapies presumably driven by ICM diagnosis or if syncope patients would tend to present less frequently at an acute care setting regardless of ICM status.

## 5. Conclusions

Among elderly patients with an ICM placement for unexplained syncope, the use of diagnostic testing during a 2-year follow-up was significantly lower compared with the pre-ICM rates, and more than one in four patients had a procedural intervention. Acute care visits for recurrent syncope were also lower during the 2 years of ICM follow-up relative to the year pre-ICM, potentially driven by the high rates of therapeutic interventions. Further studies are needed to prospectively assess the impact of ICM vs. short-term monitoring on patient outcomes and healthcare utilization.

## Figures and Tables

**Table 1:** Resultant therapy after ICM placement.

Therapy	*n* (%)	Median Time to Intervention (25–75th) (Days)	Min. (Days)	Max. (Days)
Ablation	86 (4.0%)	165 (75–348)	4.0	723.0
Pacemaker	528 (24.7%)	126 (49–324)	1.0	729.0
AICD	32 (1.5%)	236 (101–503)	1.0	725.0
Any	606 (28.3%)	127 (50–324)	1.0	729.0

## Data Availability

The data that support the findings of this analysis are available from Optum, but restrictions apply to the availability of these data, which were used under license for the current study and so are not publicly available.

## Supplementary Materials

The following supporting information can be downloaded at https://www.mdpi.com/article/10.3390/diagnostics12081977/s1: Table S1: Exclusion criteria. Table S2: Syncope-related injuries. Table S3: Baseline characteristics. Table S4: Diagnostic test utilization and repetition during the 2 years of follow-up post-ICM. Table S5: Diagnostic test utilization during the pre-ICM period. Comparison between patients with vs. without syncope events during the pre-ICM period. Table S6: Diagnostic test utilization during the 1st year of follow-up. Comparison between patients with vs. without syncope events during the pre-ICM period. Table S7: Diagnostic test utilization during the 2nd year of follow-up. Comparison between patients with vs. without syncope events during the pre-ICM period. Figure S1: Sensitivity analysis.
