# Supplementary material for: Syncope Recurrence and Downstream Diagnostic Testing after Insertable Cardiac Monitor Placement for Syncope
Source: Diagnostics (Basel). 2022 Aug 16;12(8):1977. doi: 10.3390/diagnostics12081977 (PMC9407126; doi:10.3390/diagnostics12081977)
Supplement: Supplementary file 1 [file diagnostics-12-01977-s001.zip › diagnostics-1849065-supplementary.pdf]

Supplementary Materials

Table S1: Exclusion criteria

**ICD-9 diagnosis code**	**Corresponding ICD-10 diagnosis code**	**Description**
281.1, 280.8	D508	Other iron deficiency anemias
280.9	D509	Iron deficiency anemia, unspecified
281.0	D510	Vitamin B12 deficiency anemia due to intrinsic factor deficiency
285.9	D649	Anemia, unspecified
250.11, 250.31	E101	Insulin-dependent diabetes mellitus with ketoacidosis
250.41	E102	Insulin-dependent diabetes mellitus with renal complications
250.51, 362.0x, 366.41	E103	Insulin-dependent diabetes mellitus with ophthalmic complications
250.61, 349.89, 353.5, 355.9, 357.2, 536.3	E104	Insulin-dependent diabetes mellitus with neurological complications
250.71, 443.81, 785.4	E105	Insulin-dependent diabetes mellitus with peripheral circulatory complications
250.31, 250.61, 250.81, 523.8, 713.5, 716.80	E106	Insulin-dependent diabetes mellitus with other specified complications
250.01	E109	Insulin-dependent diabetes mellitus without complications
250.20	E110	Non-insulin-dependent diabetes mellitus with coma
250.10, 250.30	E111	Non-insulin-dependent diabetes mellitus with ketoacidosis

250.40	E112	Non-insulin-dependent diabetes mellitus with renal complications
250.50, 362.0x, 366.41	E113	Non-insulin-dependent diabetes mellitus with ophthalmic complications
250.60, 349.89, 353.5, 355.9, 357.2, 536.3	E114	Non-insulin-dependent diabetes mellitus with neurological complications
250.70, 443.81, 785.4	E115	Non-insulin-depend diabetes mellitus with peripheral circulatory complications
250.30, 250.60, 250.80, 523.8, 713.5, 716.80	E116	Non-insulin-depend diabetes mellitus with other specified complications
250.90	E118	Non-insulin-dependent diabetes mellitus with unspecified complications
250.00	E119	Non-insulin-depend diabetes mellitus without complication
249.00, 250.00	E139	Other specified diabetes mellitus without complications
250.60	E144	Unspecified diabetes mellitus with neurological complications
250.00	E149	Unspecified diabetes mellitus without complications
276.50, 276.51, 276.52	E86X	Volume depletion
276.0	E870	Hyperosmolality and hypernatremia
276.1	E871	Hypo-osmolality and hyponatremia
291.x, 303.0x, 300.9x, 305.0x	F10	Mental & behavioral disorder due to use of alcohol
292.x, 304.0x, 305.5x	F11	Mental & behavioral disorder due to use of opioids

292.1x, 292.2, 292.81, 292.89, 292.9, 305.2x	F121	Mental & behavioral disorder due use cannabinoids; harmful use
292.0, 292.11, 292.12,292.2, 292.8x, 292.9, 304.1x	F132	Mental & behavioral disorder due use sedatives/hypnotics: dependence syndrome
292.11, 292.12,292.2, 292.8x, 292.9, 305.6x	F141	Mental & behavioral disorder due use cocaine: harmful use
292.0, 292.11, 292.12,292.2, 292.8x, 292.9, 304.2x, 305.60	F142	Mental & behavioral disorder due use cocaine: dependence syndrome
292.11, 292.12,292.2, 292.8x, 292.9, 305.70	F151	Mental & behavioral disorder due use other stimulant abuse including
292.0, 292.89, 292.9, 305.1	F17	Mental & behavioral disorder due use tobacco
292.11, 292.12,292.2, 292.8x, 292.9, 305.90	F189	Mental & behavioral disorder due to inhalant use, unspecified

292.11, 292.12,292.2, 292.8x, 292.9, 304.6x, 305.8x	F19	Mental & behavioral disorder multiple/psychoactive drug
332.0	G20X	Parkinson's disease (agree - not unexplained)
345.90	G40901	Status epilepticus, unspecified
491.2x, 493.2x, 496	J44x	Chronic obstruct pulmonary disorder
493.x	J45x	Predominantly allergic asthma
494.0, 494.1	J47X	Bronchiectasis
501	J61X	Pneumoconiosis due to asbestos and other mineral fibers
505	J64X	Unspecified pneumoconiosis
495	J670	Farmer's lung
495.2	J672	Bird fancier's lung
495.9	J679	Hypersensitivity pneumonitis due to unspecified organic dust
507.0	J690	Pneumonitis due to food and vomit
507.8	J698	Pneumonitis due to other solids and liquids
514, 518.4	J81X	Pulmonary edema
515, 516.3x	J841x	Other interstitial pulmonary diseases with fibrosis
516.9	J849	Interstitial pulmonary disease, unspecified
511.1, 511.89	J90X	Pleural effusion, not elsewhere classified
511.0	J920	Pleural plaque with presence of asbestos
511.0	J929	Pleural plaque without asbestos
584.9	N179	Acute renal failure, unspecified
585.6	N18.6	End-stage renal disease

586	N19X	Unspecified renal failure
078.80, 536.2, 7787.01, 787.02, 787.03, 787.04	R11X	Nausea and vomiting
646.2X, 646.4X, 646.8X, 649.5X, 649.6X, 649.7X, 679.03	O268X	Other specified pregnancy-related conditions
V53.31, V53.32, V53.39	Z450X	Adjustment and management of cardiac pacemaker
V58.81	Z452	Adjustment and management of vascular access device
V52.4, V53.99, V53.90	Z458X	Adjustment and management of other implanted devices

Table S2: Syncope-related injuries

Diagnosis codes for all-cause injury	
**ICD-9**	**ICD-10**
348.5, 733.82, 800.00, 800.50, 801.00, 801.50, 802.x, 803.00, 803.50, 830.0, 839.69, 848.0, 848.1, 848.8, 850.x, 851.x, 852.x, 853.x, 854.0x, 870.x, 871.x, 872.x, 873.x, 900.89, 905.0, 905.6, 905.7, 906.0, 906.2, 906.3, 906.4. 907.0, 907.1, 908.3, 908.9, 910.x, 918.x, 920, 921.x, 950.x, 951.x, 959.01, 959.09, V54.19, V58.89	S00 - S09 - Injuries to the head
805.0x, 805.1x, 807.5, 839.0.x, 847.0, 847.9, 848.2, 874.x, 900.x, 905.1, 905.6, 905.7, 906.0, 906.1, 906.2, 906.3, 906.4, 907.2, 907.3, 908.3, 908.9, 910.x, 920, 925.2, 952.x, 953.0, 953.4, 954.0, 957.x, 959.09, V54.17, V54.19, V58.89	S10 - S19 - Injuries to the neck
807.2, 807.3, 807.4, 805.2, 805.3, 807.0x, 807.1x, 809.0, 809.1, 839.21, 839.40, 847.1, 847.9, 848.4x, , 848.3, 860.0, 860.2, 860.4, 861.0x, 861.2x, 862.x, 875.x, 876.x, 879.0, 879.1, 901.x, 905.1, 905.6, 905.7, 906.0, 906.2, 906.3, 906.4, 907.2, 907.3, 908.0,	S20 - S29 - Injuries to the thorax

908.4, 908.9, 911.x, 922.0, 922.1, 922.33, 922.9, 926.19, 952.x, 953.1, 954.x, 957.9, 959.11, V54.17, V54.19, V58.89	
733.82, 805.4, 805.5, 805.6, 805.7, 808.x, 839.20, 839.41, 839.42, 839.49, 839.69, 846.0, 846.1, 846.9, 847.2, 847.9, 848.5, 863.2x, 863.4x, 863.8x, 863.0, 864.0x, 865.0x, 866.0x, 867.x, 868.0x, 868.13, 868.14, 869.0, 876.0, 876.1, 877.0, 877.1, 878.x, 879.x, 902.x, 905.1, 905.6, 905.7, 906.0, 906.2, 906.3, 906.4, 907.2, 907.3, 908.x, 911.x, 922.2, 922.31, 922.32, 922.4, 926.0, 926.11, 952.2, 952.3, 952.4, 953.2, 953.3, 953.5, 954.x, 959.1x, V54.13, V54.17, V54.19, V58.89,	S30 - S39 - Injuries to the abdomen, lower back, lumbar spine, pelvis and external genitals
733.81, 733.82, 810.00, 810.01, 810.02, 810.03, 810.10, 810.11, 810.12, 810.13, 811.x, 812.x, 831.0x, 839.61, 840.x, 880.x, 884.x, 887.2, 887.4, 903.x, 905.2, 905.6, 905.7, 905.9, 906.1, 906.2, 906.3, 906.4, 907.4, 908.3, 908.9, 912.x, 923.0x, 927.0x,	S40 - S49 - Injuries to the shoulder and upper arm

927.8, 927.9, 955.x, 957.9, 959.2, V54.11, V54.19, V58.89	
733.81, 733.82, 813.x, 832.x, 841.x, 881.00, 881.01, 881.10, 881.11, 881.20, 887.0, 887.2, 903.2, 903.3, 903.8, 903.9, 905.2, 905.6, 905.7, 905.9, 906.1, 906.2, 906.3, 906.4, 907.4, 908.3, 908.9, 913.x, 923.10, 923.11, 927.10, 927.11, 955.x, 957.9, 959.3, V54.10, V54.12, V58.89	S50 - S59 - Injuries to the elbow and forearm
733.81, 733.82, 814.x, 815.x, 816.x, 817.0, 818.1, 833.0x, 834.00, 834.01, 834.02, 842.x, 881.02, 881.12, 881.22, 882.0, 882.1, 882.2, 883.0, 883.1, 885.0, 886.0, 887.0, 903.2, 903.3, 903.4, 903.5, 903.8, 903.9, 905.2, 905.6, 905.7, 905.9, 906.1, 906.2, 906.3, 906.4, 907.4, 908.3, 908.9, 913.x, 914.x, 915.x, 923.20, 923.21, 923.3, 927.20, 927.21, 927.3, 955.x, 957.9, 959.3, 959.4, 959.5, V54.12, V58.89	S60 - S69 - Injuries to the wrist, hand and fingers

733.81, 733.82, 820.x, 821.x, 835.0x, 843.x, 890.x, 894.1, 897.2, 897.4, 904.x, 905.3, 905.4, 905.6, 905.7, 905.9, 906.1, 906.2, 906.3, 906.4, 907.5, 908.3, 908.9, 916.x, 924.00, 924.01, 924.4, 924.5, 928.00, 928.01, 928.8, 928.9, 929.0, 929.9, 956.x, V54.13, V54.15, V58.89	S70 - S79 - Injuries to the hip and thigh
733.81, 733.82, 822.0, 822.1, 823.x, 824.x, 827.0, 827.1, 836.x, 844.x, 845.09, 891.x, 897.0, 897.2, 897.4, 904.5x, 904.3, 904.41, 904.42, 904.7, 904.8, 905.4, 905.6, 905.7, 905.9, 906.1, 906.2, 906.3, 906.4, 907.5, 908.3, 908.9, 916.x, 924.10, 924.11, 928.10, 928.11, 956.x, 957.9, 959.7, V54.16, V58.89	S80 - S89 - Injuries to the knee and lower leg
733.81, 733.82, 825.x, 826.0, 826.1, 837.0, 838.0x, 845.x, 891.0, 891.1, 891.2, 892.0, 892.1, 893.0, 893.1, 895.0, 896.0, 904.6, 904.7, 904.8, 905.4, 905.6, 905.7, 905.9, 906.1, 906.2, 906.3, 906.4, 907.5, 908.3, 908.9, 916.x, 917.x, 924.20, 924.21, 924.3, 928.20, 928.21, 928.3, 956.3, 956.4, 956.5, 956.8, 956.9, 957.9, 959.7, V54.16, V58.89	S90 - S99 - Injuries to the head

959.8	T07 - Injuries involving multiple body regions
924.8, 959.9	T14 - Injuries to unspecified body region

Table S3: Baseline characteristics

Baseline Patient Characteristics	
Patients (N)	2140
Age, median (25th, 75th)	73 (62.0, 81.0)
Sex (% Female)	1156 (54%)
Region	
Midwest	571 (26.7%)
Northeast	168 (7.9%)
South	805 (37.6%)
West	577 (27.0%)
Unknown	19 (0.9%)
Insurance (% Medicare)	1427 (66.7%)
Syncope-related injury	396 (18.5%)
**Comorbidities**	
Hypertension	1664 (77.8%)
Diabetes	536 (25.0%)
COPD / Asthma	322 (15.1%)
Congestive Heart Failure	270 (12.6%)
Coronary Artery Disease	818 (38.2%)

Seizures	307 (14.4%)
Peripheral Artery Disease	195 (9.1%)
Transient ischemic Attack	269 (12.6%)
Charlson Comorbidity Index, Median (25th, 75th)	2.0 (1.0, 3.0)
0	533 (24.9%)
1	527 (24.6%)
2	376 (17.6%)
≥3	704 (32.9%)

Table S4: Diagnostic test utilization and repetition during 2 years of follow-up post-ICM

Diagnostic tests	Patients with ≥1 test during follow-up (N = 2,140)	Patients with repeat (≥2) tests during follow-up (N = 2,140)
Wearable ECG/Holter	64 (3.0%)	10 (0.5%)
Mobile Cardiovascular Telemetry (MCT)	13 (0.6%)	4 (0.2%)
Extended Holter	1 (0.0%)	0 (0.0%)
External loop recorder (ELR/AEM)	39 (1.8%)	8 (0.4%)
Electrocardiogram (ECG)	1,824 (85.2%)	1,524 (71.2%)
Tilt Table Testing	97 (4.5%)	27 (1.3%)
MRI (Cardiac)	15 (0.7%)	2 (0.1%)
MRI (Brain)	265 (12.4%)	100 (4.7%)
CT (Cardiac)	30 (1.4%)	5 (0.2%)
CT (Brain)	761 (35.6%)	458 (21.4%)
Electrophysiological (EP) Studies	166 (7.8%)	83 (3.9%)
Signal-averaged ECG (SAECG)	4 (0.2%)	1 (0.05%)
Electroencephalogram (EEG)	88 (4.1%)	46 (2.1%)
Carotid Doppler	487 (22.8%)	187 (8.7%)
Coronary angiogram	181 (8.5%)	97 (4.5%)
Exercise MIBI (Cardiac Stress Test)	522 (24.4%)	293 (13.7%)

Diagnostic tests	Patients with ≥1 test during follow-up (N = 2,140)	Patients with repeat (≥2) tests during follow-up (N = 2,140)
Basic laboratory testing **	1,834 (85.7%)	848 (39.6%)
Echocardiography	968 (45.2%)	466 (21.8%)

** Basic Metabolic Panel, Comprehensive Metabolic Panel, General health panel, Electrolyte panel, and their component parts

Table S5. Diagnostic test utilization during pre-ICM period – Comparison between patients with vs. without syncope events during pre-ICM period

Diagnostic tests	Patients with syncope events during pre-ICM period (N = 885)	Patients without syncope events during pre-ICM period (N = 1255)	P-value
Wearable ECG/Holter	191 (21.6%)	348 (27.7%)	0.0013*
Mobile Cardiovascular Telemetry (MCT)	92 (10.4%)	117 (9.3%)	0.4104
Extended Holter	6 (0.7%)	8 (0.6%)	0.9088
External loop recorder (ELR/AEM)	123 (13.9%)	199 (15.9%)	0.2121
Electrocardiogram (ECG)	863 (97.5%)	1,102 (87.8%)	<0.0001*
Tilt Table Testing	103 (11.6%)	109 (8.7%)	0.0243*
MRI (Cardiac)	19 (2.1%)	21 (1.7%)	0.4257
MRI (Brain)	316 (35.7%)	277 (22.1%)	<0.0001*
CT (Cardiac)	8 (0.9%)	18 (1.4%)	0.2701
CT (Brain)	651 (73.6%)	411 (32.7%)	<0.0001*
Electrophysiological (EP) Studies	67 (7.6%)	49 (3.9%)	0.0002*
Signal-averaged ECG (SAECG)	2 (0.2%)	4 (0.3%)	1.0000
Electroencephalogram (EEG)	56 (6.3%)	33 (2.6%)	<0.0001*

Diagnostic tests	Patients with syncope events during pre-ICM period (N = 885)	Patients without syncope events during pre-ICM period (N = 1255)	P-value
Carotid Doppler	429 (48.5%)	341 (27.2%)	<0.0001*
Coronary angiogram	107 (12.1%)	70 (5.6%)	<0.0001*
Exercise MIBI (Cardiac Stress Test)	374 (42.3%)	447 (35.6%)	0.0019*
Basic laboratory testing **	748 (84.5%)	1,001 (79.8%)	0.0050*
Echocardiography	724 (81.8%)	776 (61.8%)	<0.0001*

* Significant level: P<0.05

** Basic Metabolic Panel, Comprehensive Metabolic Panel, General health panel, Electrolyte panel, and their component parts

Table S6. Diagnostic test utilization during 1st year of follow-up - Comparison between patients with vs. without syncope events during pre-ICM period

Diagnostic tests	Patients with syncope events during pre-ICM period (N = 885)	Patients without syncope events during pre-ICM period (N = 1255)	P-value
Wearable ECG/Holter	15 (1.7%)	20 (1.6%)	0.8556
Mobile Cardiovascular Telemetry (MCT)	5 (0.6%)	2 (0.2%)	0.1333
Extended Holter	0 (0.0%)	0 (0.0%)	NA
External loop recorder (ELR/AEM)	11 (1.2%)	13 (1.0%)	0.6542
Electrocardiogram (ECG)	657 (74.2%)	895 (71.3%)	0.1358
Tilt Table Testing	32 (3.6%)	41 (3.3%)	0.6615
MRI (Cardiac)	4 (0.5%)	5 (0.4%)	1.0000
MRI (Brain)	70 (7.9%)	95 (7.6%)	0.7716
CT (Cardiac)	5 (0.6%)	13 (1.0%)	0.2401
CT (Brain)	239 (27.0%)	249 (19.8%)	0.0001*
Electrophysiological (EP) Studies	45 (5.1%)	89 (7.1%)	0.0591
Signal-averaged ECG (SAECG)	0 (0.0%)	0 (0.0%)	NA
Electroencephalogram (EEG)	33 (3.7%)	32 (2.5%)	0.1176

Diagnostic tests	Patients with syncope events during pre-ICM period (N = 885)	Patients without syncope events during pre-ICM period (N = 1255)	P-value
Carotid Doppler	136 (15.4%)	152 (12.1%)	0.0298*
Coronary angiogram	47 (5.3%)	48 (3.8%)	0.1002
Exercise MIBI (Cardiac Stress Test)	113 (12.8%)	187 (14.9%)	0.1618
Basic laboratory testing **	734 (82.9%)	916 (73.0%)	<0.0001*
Echocardiography	245 (27.7%)	353 (28.1%)	0.8217

* Significant level: P<0.05

** Basic Metabolic Panel, Comprehensive Metabolic Panel, General health panel, Electrolyte panel, and their component parts

Table S7. Diagnostic test utilization during 2nd year of follow-up - Comparison between patients with vs. without syncope events during pre-ICM period

Diagnostic tests	Patients with syncope events during pre-ICM period (N = 885)	Patients without syncope events during pre-ICM period (N = 1255)	P-value
Wearable ECG/Holter	13 (1.5%)	19 (1.5%)	0.9327
Mobile Cardiovascular Telemetry (MCT)	1 (0.1%)	6 (0.5%)	0.2508
Extended Holter	1 (0.1%)	0 (0.0%)	0.4136
External loop recorder (ELR/AEM)	4 (0.5%)	13 (1.0%)	0.1474
Electrocardiogram (ECG)	598 (67.6%)	797 (63.5%)	0.0519
Tilt Table Testing	10 (1.1%)	15 (1.2%)	0.8899
MRI (Cardiac)	1 (0.1%)	5 (0.4%)	0.4106
MRI (Brain)	61 (6.9%)	67 (5.3%)	0.1355
CT (Cardiac)	4 (0.5%)	9 (0.7%)	0.5762
CT (Brain)	230 (26.0%)	228 (18.2%)	<0.0001*
Electrophysiological (EP) Studies	12 (1.4%)	32 (2.5%)	0.0553
Signal-averaged ECG (SAECG)	0 (0.0%)	4 (0.3%)	0.1472
Electroencephalogram (EEG)	16 (1.8%)	18 (1.4%)	0.4960

Diagnostic tests	Patients with syncope events during pre-ICM period (N = 885)	Patients without syncope events during pre-ICM period (N = 1255)	P-value
Carotid Doppler	128 (14.5%)	159 (12.7%)	0.2304
Coronary angiogram	33 (3.7%)	62 (4.9%)	0.1803
Exercise MIBI (Cardiac Stress Test)	120 (13.6%)	162 (12.9%)	0.6611
Basic laboratory testing **	722 (81.6%)	919 (73.2%)	<0.0001*
Echocardiography	257 (29.0%)	329 (26.2%)	0.1490

* Significant level: P<0.05

** Basic Metabolic Panel, Comprehensive Metabolic Panel, General health panel, Electrolyte panel, and their component parts

Figure S1: Sensitivity analysis. Diagnostic tests used in attempt of clinical diagnosis (patients with syncope events during pre-ICM period; N=885)

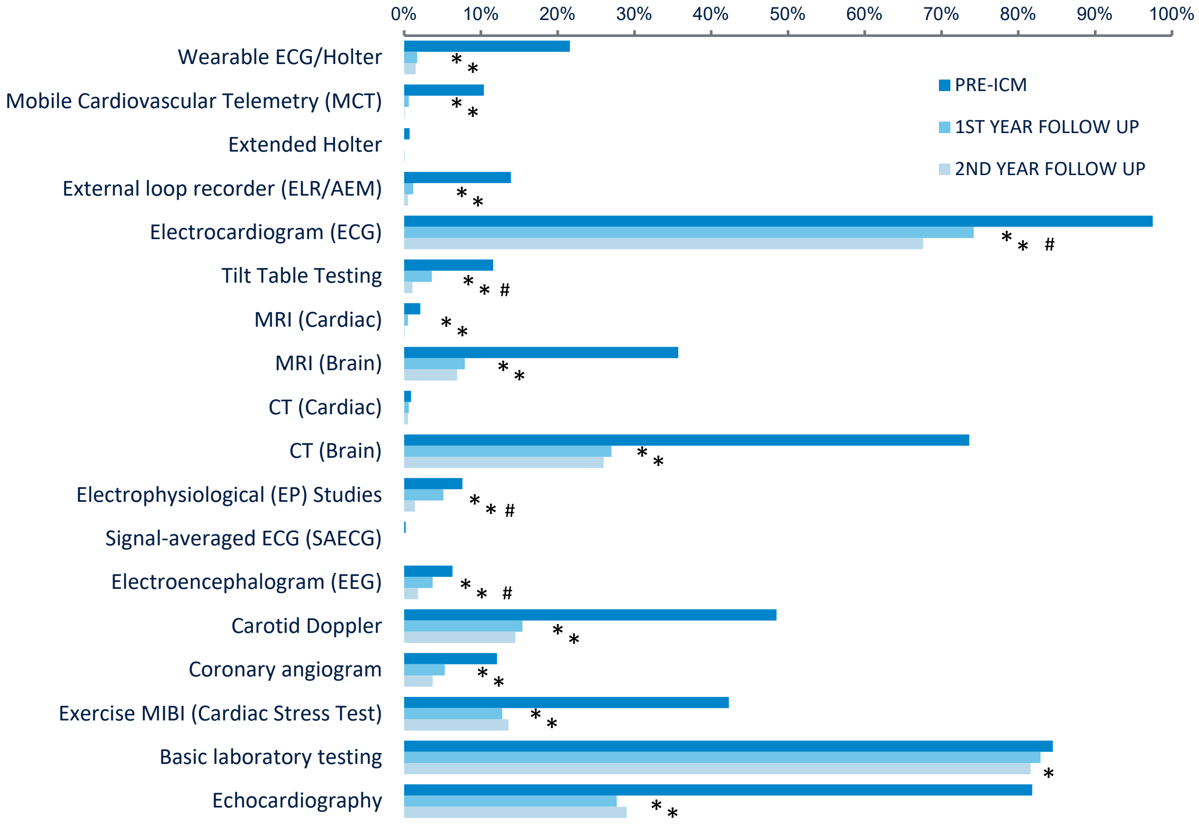

* $p<0.05$ when 1st year or 2nd year follow-up were compared to pre-ICM

# $p<0.05$ when 2nd year of follow-up was compared to 1st year follow-up
